# Iodine-131 Metaiodobenzylguanidine Therapy for Neuroblastoma: Reports So Far and Future Perspective

**DOI:** 10.1155/2015/189135

**Published:** 2015-03-22

**Authors:** Daiki Kayano, Seigo Kinuya

**Affiliations:** Department of Nuclear Medicine, Kanazawa University Hospital, 13-1 Takara-machi, Kanazawa, Ishikawa 920-8641, Japan

## Abstract

Neuroblastoma, which derives from neural crest, is the most common extracranial solid cancer in childhood. The tumors express the norepinephrine (NE) transporters on their cell membrane and take in metaiodobenzylguanidine (MIBG) via a NE transporter. Since iodine-131 (I-131) MIBG therapy was firstly reported, many trails of MIBG therapy in patients with neuroblastoma were performed. Though monotherapy with a low dose of I-131 MIBG could achieve high-probability pain reduction, the objective response was poor. In contrast, more than 12 mCi/kg I-131 MIBG administrations with or without hematopoietic cell transplantation (HCT) obtain relatively good responses in patients with refractory or relapsed neuroblastoma. The combination therapy with I-131 MIBG and other modalities such as nonmyeloablative chemotherapy and myeloablative chemotherapy with HCT improved the therapeutic response in patients with refractory or relapsed neuroblastoma. In addition, I-131 MIBG therapy incorporated in the induction therapy was proved to be feasible in patients with newly diagnosed neuroblastoma. To expand more the use of MIBG therapy for neuroblastoma, further studies will be needed especially in the use at an earlier stage from diagnosis, in the use with other radionuclide formations of MIBG, and in combined use with other therapeutic agents.

## 1. Introduction

Neuroblastoma derives from neural-crest tissues and arises mostly from adrenal medulla or paraspinal ganglia. The tumor is the most common extracranial solid cancer in childhood. The annual incidence is 10.2 cases per million children under 15 years of age [1]. More than one-third of the patients are diagnosed younger than one-year-old and the median age at diagnosis is 17 months [2]. More than half of patients have metastases at diagnosis. Main metastatic sites are regional lymph nodes, liver, bone, and bone marrow [3]. Age, stage, and MYCN status are considered as consensus determinants of prognosis. Age greater than 12 or 18 months at diagnosis and patients with an advanced primary lesion or metastases and patients with MYCN amplification have worse outcomes [2–4]. Five-year survival rates of neuroblastoma have remained approximately over 80% for infants and improved for older children from approximately 40% before 1985 to 65% in around 2000 [5]. Nevertheless, the prognosis of high-risk patients with neuroblastoma remains poor in spite of forcible multimodality therapies.

Since metaiodobenzylguanidine (MIBG) was reported as the adrenomedullary imaging agent in the early 1980s [6–8], iodine-131 (I-131) MIBG and iodine-123 (I-123) MIBG were widely used for detecting neuroendocrine tumors such as pheochromocytoma, neuroblastoma, and medullary thyroid cancer [9]. Because of emitting a beta ray with cytocidal effects, I-131 MIBG was used with the aim of treatment for neuroendocrine tumors from early after the development of MIBG. The first therapy with I-131 MIBG was applied to pheochromocytoma patients [10]. In 1986, I-131 MIBG therapy for neuroblastoma was reported for the first time [11]. Since then, many trials of I-131 MIBG therapy in patients with neuroblastoma have been done.

In this paper, we detail the development of I-131 MIBG therapy in patients with neuroblastoma from the last decades to the future.

## 2. Mechanism of MIBG Uptake in Neuroblastoma Cells

MIBG is an aralkylguanidine which is structurally similar to the neurotransmitter norepinephrine (NE) and the ganglionic blocking drug guanethidine. The uptake of MIBG in neuroendocrine cells such as normal adrenomedullary cells, neuroblastoma, and pheochromocytoma cells is similar to the uptake of NE. MIBG enters neuroendocrine cells by two pathways, a specific uptake system (uptake-one) and a nonspecific uptake system. Uptake-one is an active process via a NE transporter and is energy-requiring, sodium-dependent, temperature-dependent, and low-capacity and has a high affinity for MIBG. The nonspecific uptake is an energy-independent passive diffusional mechanism [12–14]. In the clinical setting, uptake-one is the predominant uptake system for MIBG [15, 16].

Once taken up into neuroendocrine cells, the majority of MIBG remains within the cells. MIBG is not decomposed by enzymes and is not bound to postsynaptic adrenergic receptors [17]. Most neuroendocrine cells like pheochromocytoma cells store MIBG in the neurosecretory granules. By contrast, neuroblastoma cells typically have a paucity of the neurosecretory granules and most MIBGs are stored in the cytoplasm and mitochondria, rather than in the neurosecretory granules [18, 19].

## 3. Indications and Contraindications

The indications and contraindications of I-131 MIBG therapy for neuroblastoma are stated in the European Association of Nuclear Medicine procedure guidelines [20]. The indication is Stage III or IV neuroblastoma with MIBG-avid lesions at diagnostic I-123 MIBG or I-131 MIBG scintigraphy before I-131 MIBG therapy. Because neuroblastoma arises from neural-crest tissues, most lesions express NE transporters on their cell surfaces and they take in and store radiolabeled MIBG. If radiolabeled MIBG does not accumulate in the lesions of neuroblastoma at pretherapy diagnostic study, I-131 MIBG therapy should not be performed. The aims of I-131 MIBG therapy are to achieve complete remission, to inhibit tumor progression, and to alleviate symptoms from primary or metastatic lesions. Absolute contraindications are renal failure requiring dialysis and expected life less than 3 months unless in case of refractory bone pain. Relative contraindications are provided as uncontrollable medical risk and urinary incontinence by isolation and decreased renal function by glomerular filtration rate (GFR) less than 30 mL/min.

In the guideline draft of I-131 MIBG therapy for neuroblastoma from our country, life expectancy less than not 3 months but one month and decreased renal function by GFR less than 30 mL/min are defined as absolute contraindications [21].

## 4. Toxicity of I-131 MIBG Therapy

Typical acute toxicities usually seen within two or three days after I-131 MIBG administration are nausea and vomiting. These toxicities occur in approximately 10 to 20% of treated patients. In a recent report, nausea and vomiting are observed in 11% and 21% of 66 therapies treated with upfront I-131 MIBG therapy at a dose of 4.2 to 21.7 mCi/kg for newly diagnosed neuroblastoma [22]. Sialadenitis is seen with a relatively high frequency. Five of 10 patients (9 neuroblastoma and 1 malignant pheochromocytoma) had bilateral parotid swelling within 24 hours after 12 to 18 mCi/kg I-131 MIBG injections [23].  Table 1 shows acute toxicities in 40 patients with refractory or relapsed neuroblastoma treated with I-131 MIBG at a mean dose of 10.5 mCi/kg in our institution. Though anorexia, nausea, and sialadenitis are seen with a relatively high frequency, severe acute toxicities are rare. A recent study investigated blood pressure (BP) changes within 48 hours after I-131 MIBG infusion [24]. BP-related adverse events were seen in 4 of 50 patients. One of them had a hypertensive encephalopathy. Another study reported that antihypertensive drugs were required in 2.8% of 218 I-131 MIBG administrations [25]. Though clinically relevant BP changes after I-131 MIBG therapy is rare, BP changes should be monitored at least within 48 hours after I-131 MIBG injections.

The most important toxicity is hematological with dose dependency usually appears a few weeks after MIBG therapy. Hematological toxicity is more noticeable in patients with bone marrow metastases and received higher whole-body radiation doses [26]. Hematopoietic cell transplantation (HCT) was required in about one-third of patients treated with 18 mCi/kg I-131 MIBG. In contrast, all patients treated with less than 12 mCi/kg of I-131 MIBG did not need HCT [26–28]. To date, a dose of 12 mCi/kg is considered as the maximum tolerated dose of I-131 MIBG therapy without HCT. Therefore, hematopoietic cell support should be arranged when more than 12 mCi/kg of I-131 MIBG was administrated to the patient.

Venoocclusive liver disease (VOLD) is an important early complication in patients received I-131 MIBG therapy followed by myeloablative chemotherapy and HCT. The new approaches to neuroblastoma therapy (NANT) consortium reported that 6 of 22 patients had VOLDs after the therapies and an apparently high rate of VOLD was seen in the patients with a low GFR [29]. In contrast, no VOLD was seen in patients receiving double infusions of high-dose I-131 MIBG without chemotherapy [30]. The decreased clearance of the chemotherapeutic agents was considered a major cause of VOLD.

Hypothyroidism is a major late side effect, despite the use of potassium iodine for the thyroid blockage. A Dutch group investigated the late side effect on the thyroid gland after I-131 MIBG therapy [31]. At a median follow-up time of 1.4 years after I-131 MIBG therapy, 5 of 16 survivors had TSH elevations. After a median follow-up time of 15.5 years, 8 of the 16 survivors developed hypothyroidism needed with thyroxin. In addition, papillary thyroid cancers were found in 2 of 9 survivors with thyroid nodules. Despite the protection with potassium iodine, only 3 of 16 survivors maintained normal thyroid function without any thyroid nodules. The incidence of thyroid disorders is high and increases as time advances. Papillary thyroid cancers may occur with a rather high frequency.

Second malignancies without thyroid cancers arise in less than 5%. In a report from Italy, 2 leukemia, one angiomatoid fibrous histiocytoma, one schwannoma, and one rhabdomyosarcoma occurred in 119 patients with neuroblastoma after I-131 MIBG therapy [32]. The University of California, San Francisco (UCSF) group described that leukemia was observed in 3 of 95 patients with refractory neuroblastoma at 7, 11, and 12 months after I-131 therapy [33]. It was difficult to clarify the main factor of the second malignancies, because all patients received several intensive therapies including chemotherapy and I-131 MIBG therapy.

## 5. Monotherapy with I-131 MIBG

**Figure 1:**
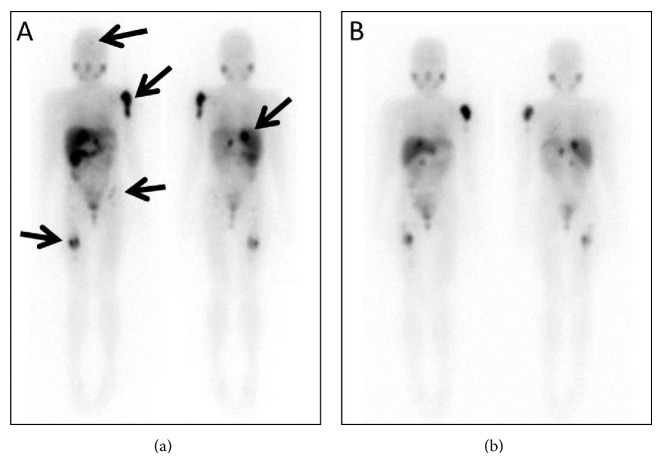
A 13-year-old female with relapsed neuroblastoma. She received the first I-131 MIBG therapy at a dose of 5.0 mCi/kg. Multiple accumulations are seen in a right retroperitoneal recurrence and multiple bone metastases ((a), arrows). The second I-131 MIBG therapy at a dose of 4.9 mCi/kg was performed 4 months after the first therapy. A scintigram after the second therapy shows a disappearance of left femoral uptake and decreasing uptakes in other lesions especially in a right retroperitoneum recurrence and a left humeral bone metastasis (b). Though the objective response at the first therapy was stable by the response evaluation criteria in solid tumors, she became free of pain in the lower extremity after the first therapy. Unfortunately, she died of progressive disease 14 months after the first I-131 MIBG therapy.

Since the first I-131 MIBG therapy for neuroblastoma were reported in 1986 [11], many monotherapy trials with I-131 MIBG for refractory or relapsed neuroblastoma were reported and obtained objective responses (partial or complete response) in 0 to 66% [27, 28, 34–42]. In a report from Germany, the objective response rate was 66% in 12 evaluable patients with refractory or relapsed neuroblastoma with a mean dose of 10.3 mCi/kg of I-131 MIBG per each therapy [37]. Compared with higher doses of I-131 MIBG, lower doses tend to achieve lower objective responses. For instance, an Italian group treated 42 patients with refractory or relapsed neuroblastoma with 75 to 162 mCi of I-131 MIBG per each therapy [39]. The objective response rate was 16.7%. Five of 7 patients with objective responses survived more than 2 years without further chemotherapy. In the phase II study by a French group, 26 patients with refractory or relapsed neuroblastoma were treated with 30 to 108 mCi of I-131 MIBG per each therapy [34]. Though pain reduction was seen in 50% of patients, no patients had any objective response. In a recent report from Israel, I-131 MIBG therapy at a dose of 5 mCi/kg (maximum dose 150 mCi) per each therapy acquired pain palliation in 90% of the first therapies and 87.5% of the second therapies in 10 symptomatic patients with refractory neuroblastoma [43]. Lower doses of I-131 MIBG obtain a few objective responses, whereas can achieve high-probability pain reduction (Figure 1).

In a phase I study from UCSF, 30 patients with refractory or relapsed neuroblastoma were treated with I-131 MIBG at escalating doses of 3 to 18 mCi/kg per each therapy [28]. The objective response rate was 37%. Most patients with objective responses were treated with 12 mCi or higher of I-131 MIBG.

A phase II study from a USA group reported some predictive factors affecting the therapeutic response of I-131 MIBG therapy in patients with refractory or relapsed neuroblastoma [27]. Sixteen patients without hematopoietic cell support were treated with 12 mCi/kg I-131 MIBG and 148 patients with hematopoietic cell support were treated with 18 mCi/kg I-131 MIBG. The overall objective response rates were 25% in patients treated with a dose of 12 mCi/kg and 37% in patients treated with a dose of 18 mCi/kg. The response rate was significantly higher in patients with fewer prior treatments, longer time from diagnosis, disease existed at soft tissue only or bone and bone marrow only and older age. The one-year event-free survival (EFS) and overall survival (OS) were 18% and 49%. The two-year OS was 29%. The EFS was significantly longer in patients with fewer prior treatments and older age.

## 6. Tandem Therapy with I-131 MIBG

Many of studies with I-131 MIBG monotherapy included patients with repetitive I-131 MIBG administrations. Each I-131 MIBG therapy was usually performed at intervals of more than 2 or 3 months because of the problems of hematologic toxicity and radiation safety.

The NANT consortium treated high-risk neuroblastoma with tandem I-131 MIBG administrations 14 days apart abrogating hematologic toxicity with autologous HCT (auto-HCT) 2 weeks after the second I-131 MIBG therapy [30]. In this dose escalation study, 20 evaluable patients received cumulative doses from 22 to 50 mCi/kg. All evaluable patients engrafted after auto-HCT and had no dose-limiting toxicity. Five of 11 patients (45.5%) with soft tissue lesions had good response. In contrast, bone marrow responses were seen in only 2 of 13 patients (15.4%). In the Children's Hospital of Philadelphia, 41 patients received repetitive I-131 MIBG therapies with auto-HCT at each dose of 18 mCi/kg [44]. The intervals of each therapy ranged from 42 to 100 days. The objective response rate after two therapies was 39%.

Though tandem therapy of I-131 MIBG with HCT is feasible, further studies are needed to improve therapeutic responses.

## 7. I-131 MIBG Therapy Combined with Chemotherapy

On the basis of I-131 MIBG monotherapeutic results, some groups tried the combination therapy with I-131 MIBG and chemotherapy agents act as radiosensitizers for refractory or relapsed neuroblastoma. In a report from Italy, 4 patients with refractory or relapsed neuroblastoma were administered I-131 MIBG in combination with cisplatin [45]. Two complete responses (CRs) and one partial response (PR) were observed. In addition, the same group treated 16 patients with 200 mCi I-131 MIBG plus cisplatin and cyclophosphamide with or without etoposide and vincristine [46]. The objective response rate was 75%. The only toxicity was hematological mainly associated with chemotherapy. Regardless of relatively low dose of I-131 MIBG, these results were superior to the reports in monotherapy trials. A group of the United Kingdom investigated the feasibility of the combination therapy with I-131 MIBG and topoisomerase I inhibitor, topotecan [47]. Eight patients were treated with 12 mCi I-131 MIBG on days 1 and 15 along with topotecan on days 1–5 and 15–19. All patients received auto-HCT on days 25–27. The combination therapy was feasible without unanticipated toxicities. The response data was not shown in the study. In a phase I study from the NANT consortium, 24 patients with refractory or relapsed neuroblastoma treated with irinotecan which is another topoisomerase I inhibitor, vincristine, and I-131 MIBG at escalating doses of 8 to 18 mCi/kg [48]. The combination therapy was well tolerated at the maximum dose of 18 mCi with controllable toxicities and then a phase II randomized study by the NANT consortium is now in progress (N2011-01). Vorinostat, a histone deacetylase inhibitor, was preclinically proved to increase expression of NE transporters in neuroblastoma cells [49]. A phase I study with a combination of I-131 MIBG and vorinostat for refractory or relapsed neuroblastoma is now examined by the NANT consortium (N2007-03).

Myeloablative chemotherapy with auto-HCT was demonstrated to improve the outcome in patients with high-risk neuroblastoma [50]. Several groups reported I-131 MIBG therapy incorporated in myeloablative chemotherapy. A pilot study from the University of Michigan examined the feasibility and efficacy of the combination therapy with I-131 MIBG and myeloablative chemotherapy in 12 patients with relapsed or advanced neuroblastoma [51]. All patients were treated with 12 mCi I-131 MIBG on day −21, followed by carboplatin, etoposide, and melphalan (CEM) administered on day −7 to day −4. Auto-HCT was performed on day 0. This regimen was well tolerated. In evaluated 8 patients, 3 CRs and 2 PRs were observed. In a phase I dose escalation study by the NANT consortium, 24 patients with refractory neuroblastoma were treated with I-131 MIBG at escalating doses of 12 to 18 mCi/kg on day −21 along with CEM on day −7 to day −4 [29]. The maximum tolerated dose of I-131 MIBG was 12 mCi/kg when received in combination of CEM in patients with normal renal function. In evaluable 22 patients, one CR and 5 PRs were observed. The estimated EFS and OS at 3 years were 0.31 ± 0.10 and 0.58 ± 0.10. A phase II study by the Children's Hospital Los Angeles using I-131 MIBG combined with CEM and auto-HCT is recently completed. The other myeloablative chemotherapy regimen using busulfan and melphalan (BuMel) with I-131 MIBG was reported form a UCSF group [52]. Eight patients with refractory neuroblastoma were treated with 18 mCi/kg I-131 MIBG on day −13 and auto-HCT on day 0. Six to eight weeks after I-131 MIBG administrations, they received busulfan on day −6 to day −2 and melphalan on day −1 and auto-HCT on day 0. I-131 MIBG therapies at doses of 18 mCi/kg were well tolerated without grade 3 or 4 nonhematologic toxicity except for one patient with sepsis. Though one patient died due to respiratory complications after the second auto-HCT, 3 CRs, and 2 PRs were observed in evaluable 7 patients. I-131 MIBG therapy with myeloablative chemotherapy followed by auto-HCT may provide additional benefit for patients with refractory or relapsed neuroblastoma. Several further studies are now ongoing.

## 8. I-131 MIBG Therapy and Allogeneic Stem Cell Transplantation

**Figure 2:**
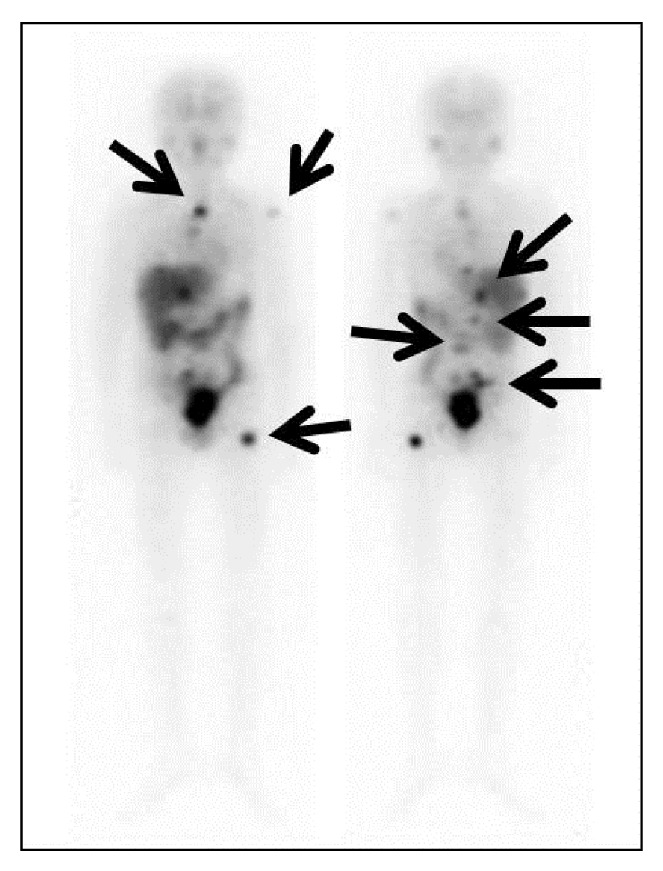
A 10-year-old male with relapsed neuroblastoma. He was treated with 16.8 mCi/kg I-131 MIBG. Multiple lymph nodes and bone metastases are detected by I-131 MIBG scintigraphy (arrows). After the treatment with chemotherapy and whole-body irradiation, he received CBSCT 4 weeks after the I-131 MIBG therapy. Complete remission has been maintained for more than 12 months.

Allogeneic HCT (allo-HCT) has been regarded as an alternative procedure for advanced neuroblastoma when autologous stem cells could not be obtained sufficiently [53, 54]. Some studies have recently reported the possibility to induce a graft-versus-tumor (GVT) effect against advanced neuroblastoma [55–57]. Two patients with relapsed neuroblastoma treated with I-131 MIBG and allo-HCT were reported in case reports from Japan [58, 59]. A 6-year-old female with relapsed neuroblastoma received reduced-intensity allo-HCT 21 days after I-131 MIBG therapy [59]. Though no GVT effect was observed, the patient was in CR for 3 months after allo-HCT. A 5-year-old female with relapsed neuroblastoma was executed cord blood stem cell transplantation (CBSCT) 9 days after I-131 MIBG therapy and GVT effect was observed after CBSCT [58]. Normalization of both vanillylmandelic acid and homovanillic acid for 5 months and decrease of I-123 MIBG accumulations were maintained, although the patient died 12 months after CBSCT. A 10-year-old male with relapsed neuroblastoma received I-131 MIBG therapy and CBSCT. Then, he got in remission for more than 12 months after the therapy (Figure 2). Though these reports indicate the potency of the combination therapy with I-131 MIBG and allo-HCT, prospective trials combining I-131 MIBG with allo-HCT will be required.

## 9. I-131 MIBG Therapy Combined with Hyperbaric Oxygen

Exposure of the neuroblastoma cells to hyperbaric oxygen (HBO) enhanced the effects of I-131 MIBG on decreasing cell proliferation and energy metabolism and increasing lipid peroxidation [60]. These effects may provide the positive effects on neuroblastoma patients treated with the combination of I-131 MIBG and HBO. A Dutch group treated 36 neuroblastoma patients with I-131 MIBG therapy alone and 27 neuroblastoma patients with a combination of I-131 MIBG therapy and 4 to 5 days HBO therapy starting 2 days after I-131 MIBG administrations [61]. The overall survival at 28 months was 32% for the I-131 MIBG and HBO combined group, compared to 12% for the group of I-131 MIBG therapy alone. Though only a few reports about the combination of I-131 MIBG and HBO therapy were shown, adding on HBO therapy may improve the effect of I-131 MIBG therapy.

## 10. I-131 MIBG Therapy for Newly Diagnosed Neuroblastoma

Based on the experience of I-131 MIBG therapy for refractory and relapsed neuroblastoma, several groups progressed to the new stage in evaluating the utility of I-131 MIBG therapy incorporated in the treatment for newly diagnosed neuroblastoma. A Dutch group used I-131 MIBG as an up-front agent of the induction therapy in patients with newly diagnosed stage IV neuroblastoma [62]. Two cycles of I-131 MIBG with a fixed dose of 200 mCi and 100 mCi were administered 4 to 6 weeks apart, followed by surgery or by both neoadjuvant chemotherapy and surgery. If objective responses were obtained after 2 cycles of I-131 MIBG therapy, patients proceeded to surgery. Some of them received additional I-131 MIBG therapies until surgery. If the objective responses were not obtained, patients were switched to induction chemotherapy until surgery. After surgery, all patients received myeloablative chemotherapy plus auto-HCT. Of the evaluable 41 patients, the objective response rate was 66% after two cycles of I-131 MIBG therapy. In addition, bone marrow metastases disappeared in 58%. Twenty-four patients received only I-131 MIBG and surgery. In the 24 patients, 14 patients had a CR after only I-131 MIBG plus surgery. The 5-year OS for all evaluated 41 patients was 14.6%. I-131 MIBG as an up-front use may be valuable for newly diagnosed advanced neuroblastoma.

In a German Neuroblastoma Trial (NB97), a benefit of I-131 MIBG therapy at the end of induction therapy in neuroblastoma patients with residual disease was investigated [63]. After induction therapy for newly diagnosed patients, 36 patients received I-131 MIBG therapy before auto-HCT and 30 patients did not receive I-131 MIBG therapy before auto-HCT. The 3-year EFS with or without I-131 MIBG therapy was 49% and 33%. The difference was not statistically significant; however these results might indicate the additive value of I-131 MIBG therapy after induction therapy in patients with newly diagnosed neuroblastoma. The following trial (NB2004) is now in progress.

An Italian group integrated I-131 MIBG therapy into induction chemotherapy in 13 patients with newly diagnosed advanced neuroblastoma [64]. In the pilot study, one CR and 6 very good PRs and 4 PRs were observed. These results support the feasibility of I-131 MIBG therapy as a part of induction therapy regimen. A pilot study of intensive induction chemotherapy and I-131 MIBG undergoing HCT for newly diagnosed advanced neuroblastoma by the Children's Oncology Group is currently recruiting participants.

Recently, a Dutch group reported the result of I-131 MIBG therapy in patients with unresectable localized neuroblastoma [65]. Twenty-one patients with any organ dysfunctions were treated with I-131 MIBG for unresectable localized neuroblastoma. Most patients needed additional surgery or both surgery and chemotherapy before or after I-131 MIBG therapy. As a result, 16 CRs, 3 very good PRs, and one PR were achieved. The 10-year EFS and OS were both 90.5%. I-131 MIBG therapy for unresectable localized neuroblastoma might be considerable when patients have any organ dysfunctions. To establish the validity and the utility of I-131 MIBG therapy for unresectable localized neuroblastoma, further studies are needed.

## 11. Other Radiopharmaceuticals in Connection with MIBG

I-131 MIBG is generally synthesized from iodine-127 MIBG by replacing stable iodine with radioiodine. Consequently, I-131 MIBG by the standard synthesis contains 1 radiolabeled I-131 MIBG molecule for 2000 nonradiolabeled MIBG molecules [66]. Nonradiolabeled MIBG competes against radiolabeled I-131 MIBG for NE transporter uptake on the cell membranes of neuroblastoma and other target organs. Some groups have synthesized no-carrier-added (NCA) I-131 MIBG and demonstrated the enhanced NCA I-131 MIBG concentrations within targets in preclinical studies [67, 68]. A phase I study about NCA I-131 MIBG showed safety in a dose escalation from 6 to 8 mCi/kg in patients with malignant pheochromocytoma or metastatic carcinoid [69]. In a phase II study by the NANT consortium, 15 patients with refractory or relapsed neuroblastoma received NCA I-131 MIBG therapy at escalation doses of 8.8 to 18.7 mCi/kg with stem cell backup [70]. Dose-limiting toxicity was not observed in all of 3, 3, and 6 patients treated with 12, 15, and 18 mCi/kg I-131 MIBG. The objective response rate was 27%, including 1 CR. NCA I-131 MIBG therapy with HCT at a dose of 18 mCi/kg is feasible without significant nonhematologic toxicity.

Because of the relatively long beta range of I-131 (0.8 mm), there is a hypothesis that I-131 fails to deliver a tumoricidal radiation dose to a small tumor less than 1 mm [71, 72]. Iodine-125 (I-125) emits very short-range Auger and conversion electrons with a high linear energy transfer and the maximum range of its emitters is about 30 *μ*m [73, 74]. Therefore, I-125 MIBG has been considered as a potential substitute for I-131 MIBG for the treatment of neuroblastoma with microscopic disease [72, 75, 76]. In phases I and II trials by the University of Michigan, 10 patients with refractory or relapsed neuroblastoma received I-125 MIBG therapy at a dose of 224 to 814 mCi [75]. The 1-year EFS was 50% and 4 patients were surviving 17 to 52 months after I-125 MIBG therapy. Further studies are needed, such as for macroscopic disease with a combination with I-125 MIBG and I-131 MIBG and for microscopic disease with I-125 MIBG.

Astatine-211 (At-211) generates alpha particles which are radiations of high linear energy transfer (LET) with very short-range in tissue of only a few cell diameters [77]. Because of shorter path length, higher LET, and more potent cytotoxicity, alpha particles are more suitable than beta particles for the targeted radionuclide therapy for microscopic disease. In a clinical experience, At-211-labeled antitenascin monoclonal antibodies were regionally administered in patients with malignant brain tumors [78]. This pilot study demonstrated the regional administration of At-211-labeled antitenascin antibody was feasible, safe, and effective for malignant brain tumors. MIBG analogue labeled with At-211, At-211 meta-astatobenzylguanidine (MABG) was proved to have a cytotoxic superiority to I-131 MIBG in human nuroblastoma cells which overexpressed NE transporters [79–81]. Alpha emitters hold enormous potentialities for radionuclide therapy. Further studies about At-211 MABG and other alpha emitters in both preclinical and clinical settings will be desired and will lead to future development of radionuclide therapy.

## 12. Conclusions

A number of studies indicate the efficacy of I-131 MIBG therapy in patients with refractory or relapsed neuroblastoma. In addition, I-131 MIBG therapy incorporated in the induction therapy is the feasible treatment strategy in patients with newly diagnosed neuroblastoma. To more expand the use of MIBG therapy for neuroblastoma, further studies will be needed especially in the use at an earlier date from diagnosis, in the use with other radionuclide formations of MIBG and in combined use with other therapeutic agents.

## Figures and Tables

**Table 1:** Acute toxicities in 40 patients with refractory or relapsed neuroblastoma treated with I-131 MIBG at a mean dose of 10.5 mCi/kg in our institution.

Toxicity	Grade (*n* = 40)			
	1	2	3	4
Anorexia	16	1	1	0
Nausea	12	1	1	0
Vomiting	2	2	0	0
Sialadenitis	2	7	0	0
Fatigue	3	1	0	0
Fever	2	1	0	0
Stomatitis	2	0	0	0

Toxicity is graded by the common terminology criteria for adverse events version 4.0.
